# Infectious Complications after Etomidate vs. Propofol for Induction of General Anesthesia in Cardiac Surgery—Results of a Retrospective, before–after Study

**DOI:** 10.3390/jcm10132908

**Published:** 2021-06-29

**Authors:** Björn Weiss, Fridtjof Schiefenhövel, Julius J. Grunow, Michael Krüger, Claudia D. Spies, Mario Menk, Jochen Kruppa, Herko Grubitzsch, Michael Sander, Sascha Treskatsch, Felix Balzer

**Affiliations:** 1 Department of Anesthesiology and Intensive Care Medicine (Campus Charité Mitte, Campus Virchow-Klinikum), Charité-Universitätsmedizin Berlin, Corporate Member of Freie Universität Berlin, Humboldt Universität zu Berlin, and Berlin Institute of Health, 13353 Berlin, Germany; bjoern.weiss@charite.de (B.W.); fridtjof.schiefenhoevel@charite.de (F.S.); julius.grunow@charite.de (J.J.G.); mikrueger@dhzb.de (M.K.); claudia.spies@charite.de (C.D.S.); mario.menk@charite.de (M.M.); 2 Institute of Health, Institute of Medical Informatics, Charité-Universitätsmedizin Berlin, Corporate Member of Freie Universität Berlin, Humboldt Universität zu Berlin, and Berlin Institute of Health, 10117 Berlin, Germany; jochen.kruppa@charite.de; 3 Department of Cardiovascular Surgery, Corporate Member of Freie Universität Berlin, Humboldt Universität zu Berlin, and Berlin Institute of Health, Charité-Universitätsmedizin Berlin, 13353 Berlin, Germany; herko.grubitzsch@charite.de; 4 Department of Anesthesiology, Intensive Care Medicine and Pain Medicine, University Hospital Gieβen, Justus-Liebig University Gieβen, 35390 Gieβen, Germany; Michael.Sander@chiru.med.uni-giessen.de; 5 Department of Anesthesiology and Operative Intensive Care Medicine (Campus Benjamin Franklin), Campus Benjamin Franklin, Charité-Universitätsmedizin Berlin, Corporate Member of Freie Universität Berlin, Humboldt Universität zu Berlin, and Berlin Institute of Health, 12203 Berlin, Germany; sascha.treskatsch@charite.de

**Keywords:** cardiac anesthesia, propofol, etomidate, infection, sepsis

## Abstract

Background: Etomidate is typically used as an induction agent in cardiac surgery because it has little impact on hemodynamics. It is a known suppressor of adrenocortical function and may increase the risk for post-operative infections, sepsis, and mortality. The aim of this study was to evaluate whether etomidate increases the risk of postoperative sepsis (primary outcome) and infections (secondary outcome) compared to propofol. Methods: This was a retrospective before–after trial (IRB EA1/143/20) performed at a tertiary medical center in Berlin, Germany, between 10/2012 and 01/2015. Patients undergoing cardiac surgery were investigated within two observation intervals, during which etomidate and propofol were the sole induction agents. Results: One-thousand, four-hundred, and sixty-two patients, and 622 matched pairs, after caliper propensity-score matching, were included in the final analysis. Sepsis rates did not differ in the matched cohort (etomidate: 11.5% vs. propofol: 8.2%, *p* = 0.052). Patients in the etomidate interval were more likely to develop hospital-acquired pneumonia (etomidate: 18.6% vs. propofol: 14.0%, *p* = 0.031). Conclusion: Our study showed that a single-dose of etomidate is not statistically associated with higher postoperative sepsis rates after cardiac surgery, but is associated with a higher incidence of hospital-acquired pneumonia. However, there is a notable trend towards a higher sepsis rate.

## 1. Introduction

Etomidate is a short-acting agonist of the GABA-A receptor that is frequently used for the induction of anesthesia in patients at high risk of hemodynamic instability. Compared with other induction agents, it is characterized by fewer hemodynamic side effects and is better able to maintain blood pressure [1]. As such, it is particularly interesting for patients at risk of severe hypotension/-perfusion or imminent cardiovascular failure [2,3]. Patients undergoing cardiac surgery are typically vulnerable to hypotension/-perfusion, which is highly undesirable because it can lead to serious adverse events [4].

Despite these potential beneficial features, etomidate is known to be a potent suppressor of adrenocortical function because it directly inhibits the biosynthesis of corticosteroids [5]. Several studies and meta-analyses have been undertaken showing that the use of etomidate as an inductive agent may be associated with complications [6,7]. For this reason, there is a consensus that etomidate should no longer be used in intensive care, or in particular, in cases of sepsis [8]. Nevertheless, it is still very commonly used in cardiothoracic anaesthesia [9]. One recently published randomized clinical trial showed that in coronary artery bypass graft (CABG) surgery with reduced ejection fraction (EF), both etomidate and propofol led to the same drop in blood pressure, which, however, could be recovered from faster in patients treated with etomidate [10]. Another randomized controlled trial revealed that propofol led to a 34% greater reduction in the mean arterial pressure (MAP) time integral compared with etomidate [1]. The authors concluded that etomidate has a superior hemodynamic profile in cardiac surgical patients.

Regarding post-acute effects, a single bolus has been shown to blunt the hypothalamic–pituitary–adrenal axis response for a prolonged period of time [11]. However, in neither the aforementioned trial nor another double-blind randomized-controlled-trial did these laboratory indicators of insufficient corticosteroid synthesis translate into increased vasopressor requirements, hemodynamic instability, or measurable adverse effects on early outcomes. Despite hemodynamic alterations and proinflammatory actions, the effect of etomidate vs. propofol on long-term trajectories is an area of current investigation [12]. Infectious complications play an extremely important role in cardiac surgery because they are a major threat to recovery [13].

We thus hypothesize that, due to the proven altered corticosteroid response, patients induced with etomidate are at greater risk of developing postoperative sepsis and infections compared with patients induced with propofol.

## 2. Materials and Methods

### 2.1. Ethics

Ethics approval was provided by the Charité—Universitätsmedizin Berlin (Charitéplatz 1, 10117 Berlin, Germany) ethics committee (EA1/143/20; chairperson: Dr. Katja Orzechowski) on the 27 July 2020.

### 2.2. Study Procedures

This single-center, retrospective, observational before–after study was conducted between October 2012 and January 2015 at a tertiary medical center in Berlin, Germany. Clinical routine data were collected from the electronic health records of the hospital and the ICU-Patient-Management-Data-System (COPRA, Sasbachwalden, Germany and SAP, Walldorf, German). All data was deidentified and all case-specific numbers were removed. The IRB waived individual informed consent for this study. The study was registered at clinical trials (CTN: NCT04281706).

### 2.3. Observed Conditions

**Figure 1:**
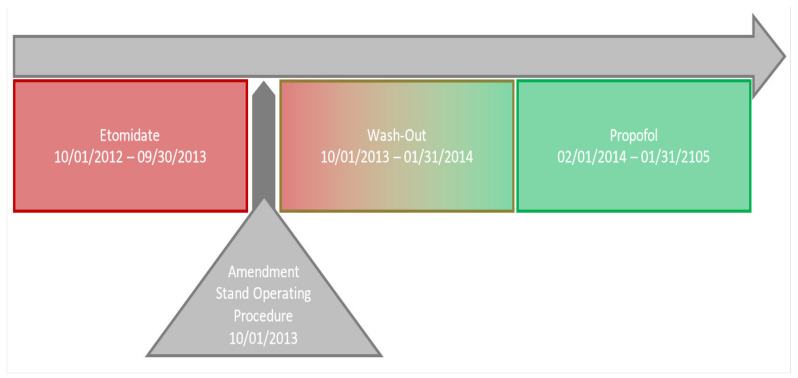
Study design. A detailed visual description of the different study periods.

The “before condition” was defined as the time period during which etomidate (eto) was used as a standard induction agent (1 October 2012–30 September 2013). The “after condition” was assessed 4 months after release of the standard operating procedure (SOP) amendment recommending propofol (prop) as the standard induction agent (1 February 2014–31 January 2015). The intervening 4-month period was considered to be a “wash-out period” when training, and thus, the SOP adherence status was likely below 100%. After 4 months, etomidate was no longer available as a medication in the anesthesia cart (Figure 1); the standard operating procedure (SOP) underwent no further amendment. Standard anesthetic practice (according to the local SOP) for patients undergoing cardiac surgery was sufentanil and cisatracurium for induction, and sevoflurane (off-pump) or propofol (on-pump) for maintenance, in combination with sufentanil. Depth of anesthesia was controlled with continuous frontal electroencephalography monitoring. In addition, all patients received standard hemodynamic monitoring and were transferred ventilated to the ICU. In the ICU, patients were treated according to a standard fast-track SOP, omitting prolonged mechanical ventilation and aiming at early extubation [14]. Patients were sedated according to the German S3-Guidelies on Analgesia, Sedation and Delirium management [8]. There was no change in practice in the postoperative treatment in the observed time period. In addition, priming of the cardiopulmonary bypass (CPB) circuit was performed in accordance with the local SOP using crystalloids, while omitting additional immunomodulatory agents, e.g., methylprednisolone.

Perioperative anti-infective prophylaxis in patients not at high risk of MRSA was conducted with a beta-lactam. The dosage was repeated every 3 h until wound-closure. Postoperatively, the prophylaxis was continued for 24 h q6 (Cefuroxim as standard). Dose adjustments according to Creatinine Clearance were made for consecutive dosages. Patients at risk of MRSA received vancomycin, 15 mg/kgIBW, in addition to the standard cephalosporine regime. All patients were equipped with a central venous-line and invasive blood pressure monitoring. Additional catheters (e.g., introducers) were only placed if medically required.

### 2.4. Patient Selection

We included 1462 patients ≥18 years undergoing valve and/or CABG surgery, and who were part of a cohort being treated in the cardiac surgery department. We excluded surgical procedures in the “wash-out” period, re-surgery, minimally invasive procedures (e.g., PM implantations), surgery because of endocarditis, patients with pre-known immunosuppression (chronic corticosteroid therapy, solid organ transplant, stem cell therapy, or HIV diagnosis), and patients with preoperative stroke. We observed patients until discharge from the hospital.

### 2.5. Measuring of Outcomes

The primary outcome was sepsis measured according to the International Classification of Diseases, Tenth Revision (ICD-10), in the electronic health record (EHR). At the time of the study, sepsis was defined and diagnosed using the Sepsis-2 criteria (≥2 Systemic inflammatory response syndrome criteria and infection or suspected infection), which were standard in the intensive care unit during the observation period.

Secondary outcomes, such as the incidence of hospital-acquired infections (HAIs), were derived from the ICD-10 coding for pneumonia (HAP), *Clostridium difficile* associated diarrhea (CDAD), superficial and deep wound infections, and urinary tract infections that were documented in patients’ EHR. Data regarding mortality and length of stay (LOS) in the ICU and hospital, and ventilation time, were derived from the EHR. As for the primary outcome, we observed patients’ until discharge from hospital.

The ICD-10 diagnoses were made on the basis of PDMS values that were regularly subject to routine monitoring.

### 2.6. Statistical Analysis

Descriptive analyses and statistical testing were performed using the R Project of Statistical Computing 3.6.2., R Core Team, Vienna, Austria (2019). When a normal distribution was ruled out using the Shapiro–Wilk test, results were listed as the median and interquartile ranges, and otherwise as the mean and standard deviation. Qualitative observations (of categorical variables) are reported by absolute and relative frequencies. Statistical significance among groups was analyzed using the t-test in the case of normally distributed variables, and using the Kruskal–Wallis test when variables were found to be non-normally distributed. Exact chi-square tests were used for qualitative data. Propensity score matching was performed for dichotomized levels of urgency, and the presence of any diagnostic code for heart insufficiency using the NYHA (New York Heart Association) classification, and APACHE 2 (Acute Physiology and Chronic Health Evaluation II) score using the R package MatchIt [15], using the nearest neighbor method, linear.logit distance, ratio 1, caliper 0.2, and m.order largest. All tests should be understood as constituting explorative analysis; no adjustment for multiple testing was made.

## 3. Results

### 3.1. Patient Characteristics and Matching

**Figure 2:**
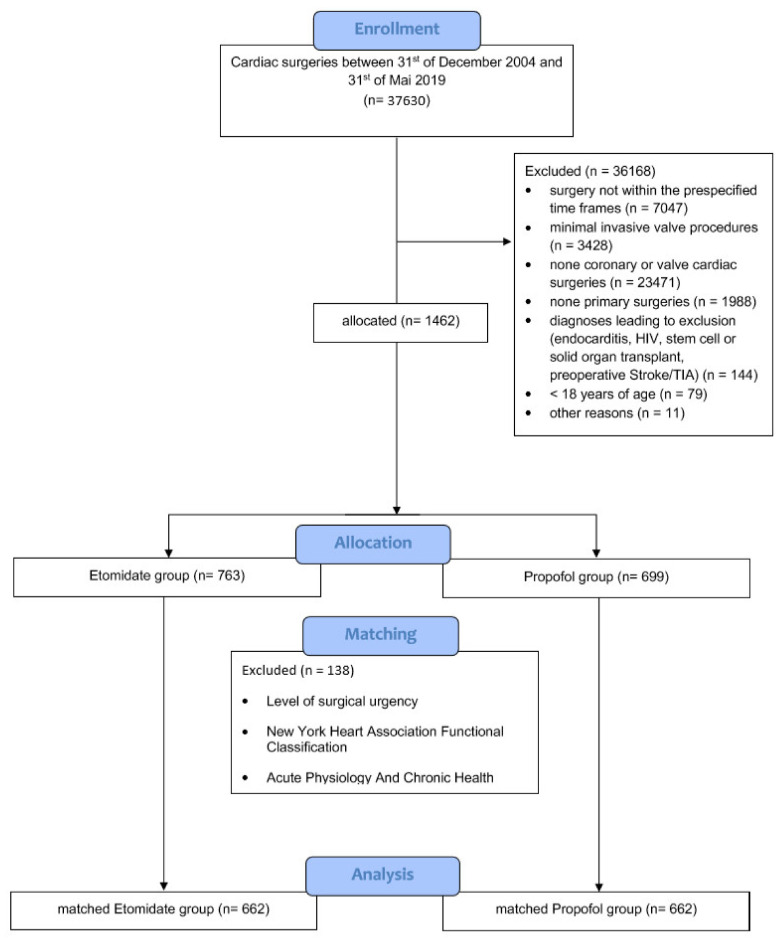
Study flow chart. A detailed visual description of the numbers of included and excluded patients.

All patients treated in the center during the study interval were screened for eligibility (*n* = 37,630). After selection based on inclusion and exclusion criteria, 1462 patients were included in the analysis (Figure 2). The basic patient characteristics of matched and unmatched cohorts are shown in Table 1. The unmatched cohort consisted of *n* = 763 patients in the etomidate group and *n* = 699 patients in the propofol group.

Patients in the propofol group had significantly higher APACHE 2 scores on admission to the ICU post-surgery (etomidate: 16.0 (11–33) vs. propofol: 18.0 (11–35), *p* = 0.002). In addition, in this group, significantly more patients underwent emergency surgery (etomidate: 13.8% vs. propofol: 19.7%, *p* = 0.003). Furthermore, there were marginal, statistically significant differences in body mass index (BMI) and ASA status (Table 1). The subjects were matched based on the dichotomized level of urgency, APACHE 2 score, and the presence of any diagnostic code for heart failure using the NYHA classification. Matching was undertaken using a caliper propensity-score approach, in which a 0.2 standard deviation was allowed as the default (caliper widths). We identified 662 matched pairs. ASA status and BMI still showed the statistical difference without clinical significance.

### 3.2. Infections

In the unmatched (etomidate: 11.4% vs. propofol: 8.4%, *p* = 0.072) and the matched cohorts (etomidate: 11.5% vs. propofol: 8.2%, *p* = 0.052), patients treated with etomidate did not have a significantly higher sepsis rate. Patients receiving etomidate were significantly more likely to develop hospital-acquired pneumonia independently of the matching (matched; etomidate: 18.6% vs. propofol: 14.0%, *p* = 0.031). Interestingly, neither surgical site infections nor other infectious complications showed any significant difference between the groups. Further details on the comparison of infectious complications are displayed in Table 2.

### 3.3. Secondary Outcomes

ICU mortality did not significantly differ between the two groups (matched, etomidate: 6.0% vs. propofol: 4.1%, *p* = 0.132). There was a statistically significant longer hospital LOS in the etomidate group (both groups stayed a median of 12 days). In contrast, etomidate patients stayed on average 22 h shorter in the ICU in the matched and unmatched cohorts (*p* < 0.001 for matched and unmatched). Postoperative stroke rates did not significantly differ between the two groups. A comprehensive summary of these outcomes can be found in Table 3.

## 4. Discussion

In this retrospective before–after trial, we found no statistically significant difference between sepsis rates in patients that were treated with etomidate vs. propofol for induction of general anesthesia. However, there was a significantly higher rate of hospital-acquired pneumonia in patients treated with etomidate vs. propofol. We found no clinically relevant differences between ICU mortality, ICU length of stay, or hospitalization time, but a 22 h longer ICU lengths-of-stay in the propofol group. Although the ventilation-time was 2 h longer in the etomidate group and this difference was statistically significant, it can be considered a clinically insignificant difference and was most likely the result of organizational reasons.

Sepsis is a highly relevant complication after cardiac surgery because it is associated with high mortality, which is reported to be between 65% and 79% [16]. However, the incidence depends on the type and mode of surgery. We experienced a comparatively high number of emergency surgeries, which explains that our rates were observed to be slightly higher than those reported in previous trials [17]. A recently published retrospective cohort study comparing etomidate with propofol found a trend towards a higher rate of infection in the etomidate group, which did not reach statistical significance [18]. Despite the barely missed significance in our study (*p* = 0.052), there was a notable trend towards a higher sepsis rate. This should be noted because the effect size is about the same as that of HAP. A clear limitation of the mentioned study in comparison to our cohort was the low number of cases (*n* = 129) and the imbalance between the control and case numbers. However, clinicians might consider this trend when choosing their induction agent.

HAP has been previously shown to be associated with the use of etomidate. In an ancillary study of the HYPOLITE RCT, Asehnoue and colleagues revealed that, in critically ill trauma patients, etomidate increased the odds of pneumonia by more than twofold [19]. This finding is in line with our results, but our effect size is smaller. Other clinical trials on etomidate focused mainly on the hemodynamic profile, safety, and cortisol suppression. A Cochrane review on the outcome effect of a single dose of etomidate for emergency airway intervention revealed no conclusive evidence that etomidate increases mortality or other clinical outcomes [20]. However, several trials show that it has an effect on adrenal function, and thus causes complications [21].

The pathophysiologic link between higher infection rates and the use of etomidate may lie in this impairment of adrenal function. Vinclair and colleagues revealed that a single bolus of etomidate is associated with a significant adrenal inhibition in critically ill patients [22]. The authors also revealed that the alteration was reversible within approximately 48 h in most patients and did not affect clinical outcomes. Nonetheless, the generalizability is limited because the study was not sufficiently powered to reveal clinical differences (*n* = 40) [22]. Another important study of risk factors for adrenocortical deficiency in critically ill patients showed that etomidate as a single dose is the most significant risk factor for relative adrenal inhibition in critically ill patients [23].

Surprisingly, most previous studies, such as those mentioned above, were not able to show a clinical disadvantage for patients receiving etomidate [20,24,25,26]. Our data are in line with these studies, as we could not show clinically relevant differences in ICU mortality or length of hospital stay [20,22,26,27] Notwithstanding the fact that there was a statistically significant trend towards a shorter hospitalization time in the propofol group, and in contrast, a 22-h longer ICU-stay in the propofol group, the changes in hospital length-of-stay might be interpreted as not clinically relevant. For the ICU length of stay, the effect was statistically significant and might also be meaningful in some clinical situations. In our setting, the discharge from the ICU to the intermediate care unit for post-cardiac surgery also depends highly on the capacity of the other unit, so a variation of one day is likely due to organizational reasons. However, future research might elaborate on that difference.

Our study has limitations that should be carefully considered when interpreting the results. Before–after cohort studies are per se non-experimental studies that underlie certain special methodological aspects. The threats to internal validity are particularly noteworthy in this context. From a statistical point of view, the used propensity score matching approach accounts for specific confounders but reduces the cohort size, and of course, cannot adjust for unknown and potentially unmeasured confounders. A randomized approach would be the method of choice in this case.

In our design, maturation and dropout threats were the most significant problems. The first factor refers to the fact that the intervention group could have developed independently from the intervention, and the latter factor refers to a change in case mix. As our peri- and postoperative management was driven by SOPs, and these were not changed, we can exclude the fundamental influences of our routine practice. However, the increased experience of staff may have also influenced the outcome parameters. In summary, we reduced maturity and dropout threats by ensuring experienced staff utilized the SOPs, with no changes, except the intervention, in a limited time frame. An additional limitation is that outcomes could only be measured during the time of hospitalization and not a predefined time scale. In particular, non-life threatening, late infectious complications might have remained unobserved.

Due to the study’s limitations, the results should be seen to be exploratory. Furthermore, sample size may have contributed to the finding of no statistical difference in sepsis rates. Randomized, prospective trials based on a sufficient sample size calculation are necessary for confirmatory analysis. In this case, the sepsis rate must not be overestimated on the basis of our data, because previous trials excluding emergency surgery found lower sepsis rates. In addition, the EuroScore, which is a potent tool for predicting outcomes, would have allowed improved detection of group imbalances, but was not available in our analysis [28].

## 5. Conclusions

In summary, our study showed that a single dose of etomidate was not statistically associated with higher postoperative sepsis rates, but was statistically associated with a higher incidence of HAP. Despite the barely missed significance (*p* = 0.052), there was a notable trend towards a higher sepsis rate. This should be noted because the effect size was the same in the two cases.

However, as reported previously, there was not a clinically relevant difference in mortality or resource use in patients receiving a single bolus etomidate compared with those receiving propofol. This might have been due to the fact that potential complications of an adrenal inhibiting effect can be treated immediately in the critical care context.

## Figures and Tables

**Table 1:** Basic characteristics of matched and unmatched cohorts.

		Unmatched				Matched (Level of Surgical Urgency, NYHA, APACHE II)			
		All (*n* = 1462)	Etomidate (*n* = 763)	Propofol (*n* = 699)	*p*-Value	All (*n* = 1324)	Etomidate (*n* = 662)	Propofol (*n* = 662)	*p*-Value
Age (years)		70.0 (61.1–35.0)	70.0 (62.1–35.5)	69.0 (61.1–35.0)	0.262	70.0 (61.1–35.0)	70.0 (62.1–35.0)	69.0 (61.1–35.8)	0.287
Sex (male/female)		1083 (74.1)/ 379 (25.9)	561 (73.5)/ 202 (26.5)	522 (74.7)/ 177 (25.3)	0.658	992 (74.9)/ 332 (25.1)	494 (74.6)/ 168 (25.4)	498 (75.2)/ 164 (24.8)	0.849
BMI (kg/m^2^)		27.5 (24.1–30.8)	27.4 (24.1–30.4)	27.7 (25.1–31.2)	0.032	27.5 (24.1–30.8)	27.4 (24.1–30.4)	27.7 (25.1–31.2)	0.037
ASA	2	31 (2.1)	15 (2.0)	16 (2.3)	0.001	26 (2.0)	12 (1.8)	14 (2.1)	0.001
	3	950 (65.0)	508 (66.6)	442 (63.2)		857 (64.7)	433 (65.4)	424 (64.0)	
	4	194 (13.3)	117 (15.3)	77 (11.0)		182 (13.7)	110 (16.6)	72 (10.9)	
	5	5 (0.3)	4 (0.5)	1 (0.1)		5 (0.4)	4 (0.6)	1 (0.2)	
	missing	282 (19.3)	119 (15.6)	162 (23.3)		254 (19.2)	103 (15.6)	151 (22.8)	
Charlson Comorbidity Index		4.0 (3.1–3.0)	4.0 (3.1–3.0)	4.0 (3.1–3.0)	0.660	4.0 (3.1–3.0)	4.0 (3.1–3.0)	4.0 (3.1–3.0)	0.796
APACHE II		17.0 (12.1–34.0)	16.0 (11.1–33.0)	18.0 (12.1–35.0)	0.002	18.0 (12.1–35.0)	17.0 (12.1–35.0)	18.0 (13.1–35.0)	0.116
NYHA any classification		708 (48.4)	404 (52.9)	304 (43.5)	<0.001	594 (44.9)	305 (46.1)	289 (43.7)	0.407
Surgery	CABG	1001 (68.5)	516 (67.6)	485 (69.4)	0.539	906 (68.4)	452 (68.3)	454 (68.6)	0.725
	Valve	325 (22.2)	170 (22.3)	155 (22.2)		294 (22.2)	144 (21.8)	150 (22.7)	
	Combined	136 (9.3)	77 (10.1)	59 (8.4)		124 (9.4)	66 (10.0)	58 (8.8)	
Surgical Mode	Elective	1219 (83.4)	658 (86.2)	561 (80.3)	0.003	1098 (82.9)	559 (84.4)	539 (81.4)	0.165
	Urgent/Emergency	243 (16.6)	105 (13.8)	138 (19.7)		226 (17.1)	103 (15.6)	123 (18.6)	
Diabetes Mellitus		564 (38.6)	287 (37.6)	277 (39.6)	0.462	501 (37.8)	240 (36.3)	261 (39.4)	0.257
Chronic Kidney Disease		344 (23.5)	192 (25.2)	152 (21.7)	0.140	318 (24.0)	171 (25.8)	147 (22.2)	0.139
Arterial Hypertension		1230 (84.1)	640 (83.9)	590 (84.4)	0.839	1113 (84.1)	554 (83.7)	559 (84.4)	0.764
Pacemaker		65 (4.5)	37 (4.9)	28 (4.0)	0.513	60 (4.5)	32 (4.8)	28 (4.2)	0.692

Values are presented as counts (percentages) or medians (interquartile ranges). Statistical analysis was performed in R, using the compareGroups package. Statistical test used: for numerical variables: Kruskal–Wallis; for categorical variables: chi-square. BMI = body mass index. ASA = American Society of Anesthesiologists Classification; APACHE = Acute Physiology And Chronic Health Evaluation; NYHA = New York Heart Association Classification; CABG = coronary artery bypass graft.

**Table 2:** Infectious complications.

	Unmatched				Matched (Level of Surgical Urgency, NYHA, APACHE II)			
	All (*n* = 1462)	Etomidate (*n* = 763)	Propofol (*n* = 699)	*p*-Value	All (*n* = 1324)	Etomidate (*n* = 662)	Propofol (*n* = 662)	*p*-Value
Sepsis	146 (10.0)	87 (11.4)	59 (8.4)	0.072	130 (9.8)	76 (11.5)	54 (8.2)	0.052
Any Infection	415 (28.4)	230 (30.1)	185 (26.5)	0.134	372 (28.1)	200 (30.2)	172 (26.0)	0.099
Surgical Wound Infection	107 (7.3)	55 (7.2)	52 (7.4)	0.945	96 (7.3)	47 (7.1)	49 (7.4)	0.916
Pneumonia	249 (17.0)	146 (19.1)	103 (14.7)	0.030	216 (16.3)	123 (18.6)	93 (14.0)	0.031
C. difficile Enterocolitis	33 (2.3)	12 (2.8)	12 (1.7)	0.248	27 (2.0)	17 (2.6)	10 (1.5)	0.243
Urinary Tract Infection	132 (9.0)	76 (10.0)	56 (8.0)	0.227	124 (9.4)	70 (10.6)	54 (8.2)	0.157

Values are presented as counts (percentages). Statistical analysis was undertaken in R, using the compareGroups package. Statistical test used: for numerical variables: Kruskal–Wallis; for categorical variables: chi-square. Complication: incidence of any postoperative infection, non-cumulative. Surgical wound infection: incidence of infection of the surgical site, non-cumulative. Pneumonia: incidence of postoperative pneumonia, non-cumulative. *C. difficile* Enterocolitis: incidence of postoperative infection with *Clostridium difficile*, non-cumulative. Sepsis: incidence of postoperative sepsis, non-cumulative. Urinary tract infection: incidence of postoperative urinary tract infection, non-cumulative.

**Table 3:** General outcome variables.

	Unmatched				Matched (Level of Surgical Urgency, NYHA, APACHE II)			
	All (*n* = 1462)	Etomidate (*n* = 763)	Propofol (*n* = 699)	*p*-Value	All (*n* = 1324)	Etomidate (*n* = 662)	Propofol (*n* = 662)	*p*-Value
ICU Mortality	75 (5.1)	46 (6.0)	29 (4.2)	0.131	67 (5.1)	40 (6.0)	27 (4.1)	0.132
Hospital Mortality	77 (5.3)	48 (6.3)	29 (4.2)	0.086	68 (5.1)	41 (6.2)	27 (4.1)	0.106
Hospital LOS of survivors (days)	12.0 (9.1–39.0)	12.0 (9.1–30.0)	12.0 (8.1–37.0)	<0.001	12.0 (9.1–38.0)	12.0 (9.1–30.0)	12.0 (8.1–37.0)	<0.001
ICU LOS of survivors (hours)	140 (91–311)	121 (91–395)	144 (111–315)	<0.001	141 (91–311)	122 (91–304)	144 (111–315)	<0.001
Ventilation (hours)	17.0 (11.0; 32.0)	18.0 (11.0; 34.0)	16.0 (10.0; 29.0)	0.012	17.00 (11.00; 32.00)	18.00 (11.00; 35.00)	16.00 (10.0; 29.0)	0.004
Stroke post Surgery	27 (1.9)	19 (2.5)	8 (1.1)	0.086	25 (1.9)	17 (2.6)	8 (1.2)	0.106

Values are presented as counts (percentages) or medians (interquartile ranges). Statistical analysis was performed in R, using the compareGroups package. Statistical test used: for numerical variables: Kruskal–Wallis; for categorical variables: chi-square. ICU = intensive care unit.

## Data Availability

The data presented in this study are available on request from the corresponding author. The data are not publicly available due to local data protection requirements.

## References

[1] Hannam J.A., Mitchell S.J., Cumin D., Frampton C., Merry A.F., Moore M.R., Kruger C.J. (2019). Haemodynamic profiles of etomidate vs propofol for induction of anaesthesia: A randomised controlled trial in patients undergoing cardiac surgery. Br. J. Anaesth..

[2] Aggarwal S., Goyal V.K., Chaturvedi S.K., Mathur V., Baj B., Kumar A. (2016). A comparative study between propofol and etomidate in patients under general anesthesia. Braz. J. Anesthesiol..

[3] Kaushal R.P., Vatal A., Pathak R. (2015). Effect of etomidate and propofol induction on hemodynamic and endocrine response in patients undergoing coronary artery bypass grafting/mitral valve and aortic valve replacement surgery on cardiopulmonary bypass. Ann. Card. Anaesth..

[4] Wesselink E.M., Kappen T.H., van Klei W.A., Dieleman J.M., van Dijk D., Slooter A.J. (2015). Intraoperative hypotension and delirium after on-pump cardiac surgery. Br. J. Anaesth..

[5] Wagner R.L., White P.F. (1984). Etomidate inhibits adrenocortical function in surgical patients. Anesthesiology.

[6] Chan C.M., Mitchell A.L., Shorr A.F. (2012). Etomidate is associated with mortality and adrenal insufficiency in sepsis: A meta-analysis. Crit. Care Med..

[7] Sunshine J.E., Deem S., Weiss N.S., Yanez N.D., Daniel S., Keech K., Brown M., Treggiari M.M. (2013). Etomidate, adrenal function, and mortality in critically ill patients. Respir. Care.

[8] DAS-Taskforce (2015). Evidence and consensus based guideline for the management of delirium, analgesia, and sedation in intensive care medicine. Revision 2015 (DAS-Guideline 2015)—short version. Ger. Med. Sci..

[9] Wagner C.E., Bick J.S., Johnson D., Ahmad R., Han X., Ehrenfeld J.M., Schildcrout J.S., Pretorius M. (2014). Etomidate use and postoperative outcomes among cardiac surgery patients. Anesthesiology.

[10] Soleimani A., Heidari N., Habibi M.R., Kiabi F.H., Khademloo M., Emami Zeydi A., Sohrabi F.B. (2017). Comparing hemodynamic responses to diazepam, propofol and etomidate during anesthesia induction in patients with left ventricular dysfunction undergoing coronary artery bypass graft surgery: A double-blind, randomized clinical trial. Med. Arch..

[11] Morel J., Salard M., Castelain C., Bayon M.C., Lambert P., Vola M., Auboyer C., Molliex S. (2011). Haemodynamic consequences of etomidate administration in elective cardiac surgery: A randomized double-blinded study. Br. J. Anaesth..

[12] Basciani R.M., Rindlisbacher A., Begert E., Brander L., Jakob S.M., Etter R., Carrel T., Eberle B. (2016). Anaesthetic induction with etomidate in cardiac surgery: A randomised controlled trial. Eur. J. Anaesthesiol..

[13] Ailawadi G., Chang H.L., O’Gara P.T., O’Sullivan K., Woo Y.J., DeRose J.J.J., Parides M.K., Thourani V.H., Robichaud S., Gillinov A.M. (2017). Pneumonia after cardiac surgery: Experience of the national institutes of health/Canadian institutes of health research cardiothoracic surgical trials network. J. Thorac. Cardiovasc. Surg..

[14] Habicher M., Zajonz T., Heringlake M., Boning A., Treskatsch S., Schirmer U., Markewitz A., Sander M. (2018). S3 guidelines on intensive medical care of cardiac surgery patients: Hemodynamic monitoring and cardiovascular system-an update. Der Anaesthesist.

[15] Ho D.E., Imai K., King G., Stuart E. (2011). MatchIt: Nonparametric Preprocessing for Parametric Causal Inference. J. Stat. Softw..

[16] Paternoster G., Guarracino F. (2016). Sepsis after cardiac surgery: From pathophysiology to management. J. Cardiothorac. Vasc. Anesth..

[17] Howitt S.H., Herring M., Malagon I., McCollum C.N., Grant S.W. (2018). Incidence and outcomes of sepsis after cardiac surgery as defined by the Sepsis-3 guidelines. Br. J. Anaesth..

[18] Hidalgo D.C., Amin V., Hukku A., Kutlu K., Rech M.A. (2020). Etomidate use for rapid sequence intubation is not associated with nosocomial infection. J. Pharm. Pract..

[19] Asehnoune K., Mahe P.J., Seguin P., Jaber S., Jung B., Guitton C., Chatel-Josse N., Subileau A., Tellier A.C., Masson F. (2012). Etomidate increases susceptibility to pneumonia in trauma patients. Intensive Care Med..

[20] Bruder E.A., Ball I.M., Ridi S., Pickett W., Hohl C. (2015). Single induction dose of etomidate versus other induction agents for endotracheal intubation in critically ill patients. Cochrane Database Syst. Rev..

[21] Albert S.G., Ariyan S., Rather A. (2011). The effect of etomidate on adrenal function in critical illness: A systematic review. Intensive Care Med..

[22] Vinclair M., Broux C., Faure P., Brun J., Genty C., Jacquot C., Chabre O., Payen J.F. (2008). Duration of adrenal inhibition following a single dose of etomidate in critically ill patients. Intensive Care Med..

[23] Malerba G., Romano-Girard F., Cravoisy A., Dousset B., Nace L., Levy B., Bollaert P.E. (2005). Risk factors of relative adrenocortical deficiency in intensive care patients needing mechanical ventilation. Intensive Care Med..

[24] Cuthbertson B.H., Sprung C.L., Annane D., Chevret S., Garfield M., Goodman S., Laterre P.F., Vincent J.L., Freivogel K., Reinhart K. (2009). The effects of etomidate on adrenal responsiveness and mortality in patients with septic shock. Intensive Care Med..

[25] Jung B., Clavieras N., Nougaret S., Molinari N., Roquilly A., Cisse M., Carr J., Chanques G., Asehnoune K., Jaber S. (2012). Effects of etomidate on complications related to intubation and on mortality in septic shock patients treated with hydrocortisone: A propensity score analysis. Crit. Care.

[26] McPhee L.C., Badawi O., Fraser G.L., Lerwick P.A., Riker R.R., Zuckerman I.H., Franey C., Seder D.B. (2013). Single-dose etomidate is not associated with increased mortality in ICU patients with sepsis: Analysis of a large electronic ICU database. Crit Care Med..

[27] Heinrich S., Schmidt J., Ackermann A., Moritz A., Harig F., Castellanos I. (2014). Comparison of clinical outcome variables in patients with and without etomidate-facilitated anesthesia induction ahead of major cardiac surgery: A retrospective analysis. Crit. Care.

[28] Nashef S.A., Roques F., Michel P., Gauducheau E., Lemeshow S., Salamon R. (1999). European system for cardiac operative risk evaluation (EuroSCORE). Eur. J. Cardiothorac. Surg..

